# During spontaneous breathing cardiac output lacks major effect on pulmonary shunting in porcine lungs with partial collapse

**DOI:** 10.1186/cc10727

**Published:** 2012-03-20

**Authors:** L Vimlati, A Larsson, G Hedenstierna, M Lichtwarck-Aschoff

**Affiliations:** 1 Uppsala University, Uppsala, Sweden

## Introduction

Spontaneous breathing (SB) improves oxygenation compared to mechanical ventilation (MV), and does so even without recruit-ing atelectasis [1,2]. Since it cannot be excluded that cardiac output (CO) impacts on pulmonary shunt, we investigated whether pulmonary shunt correlates with CO in a porcine model of lung collapse.

## Methods

In 12 anaesthetized and relaxed supine piglets, lung collapse was induced by negative pressure application to the endotracheal tube during MV. Six animals resumed SB after 15 minutes; the other six were kept on MV at a respiratory rate and tidal volume corresponding to SB. All animals were followed over 120 minutes, and repeated measurements were converted to the area under curve and analysed by Mann-Whitney test and linear regression.

## Results

**Figure 1:**
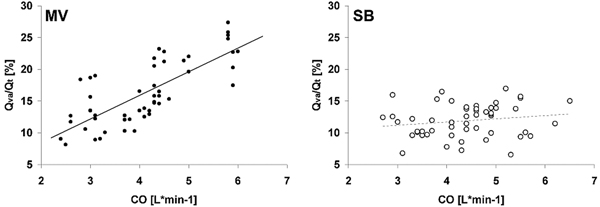
**Venous admixture (Qva/Qt) plotted against CO (pooled data for each group)**. Solid circles, mechanical ventilation (MV); open circles, spontaneous breathing (SB).

PaO_2_/FiO_2 _was higher and venous admixture (Qva/Qt) was lower in the SB group. Hemodynamics was stable and CO was similar in both groups. Qva/Qt correlated with CO (*r *= 0.83, *P *= 0.04) in the MV group, but not in the SB group (*r *= 0.08, *P *= 0.88) (Figure 1).

## Conclusion

SB achieves higher PaO_2_/FiO_2 _and lower Qva/Qt compared to MV. During SB, Qva/Qt seems to be unaffected by CO. This lung collapse model has stable hemodynamics and gas exchange for at least 2 hours irrespective of the mode of ventilation.
